# A Roadmap of CAR-T-Cell Therapy in Glioblastoma: Challenges and Future Perspectives

**DOI:** 10.3390/cells13090726

**Published:** 2024-04-23

**Authors:** Megan Montoya, Marco Gallus, Su Phyu, Jeffrey Haegelin, John de Groot, Hideho Okada

**Affiliations:** 1 Department of Neurological Surgery, University of California San Francisco, San Francisco, CA 94143, USA; 2 Helen Diller Family Comprehensive Cancer Center, San Francisco, CA 94158, USA; 3 Parker Institute for Cancer Immunotherapy, San Francisco, CA 94129, USA

**Keywords:** glioblastoma, CAR-T-cell therapy, tumor immune microenvironment

## Abstract

Glioblastoma (GBM) is the most common primary malignant brain tumor, with a median overall survival of less than 2 years and a nearly 100% mortality rate under standard therapy that consists of surgery followed by combined radiochemotherapy. Therefore, new therapeutic strategies are urgently needed. The success of chimeric antigen receptor (CAR) T cells in hematological cancers has prompted preclinical and clinical investigations into CAR-T-cell treatment for GBM. However, recent trials have not demonstrated any major success. Here, we delineate existing challenges impeding the effectiveness of CAR-T-cell therapy for GBM, encompassing the cold (immunosuppressive) microenvironment, tumor heterogeneity, T-cell exhaustion, local and systemic immunosuppression, and the immune privilege inherent to the central nervous system (CNS) parenchyma. Additionally, we deliberate on the progress made in developing next-generation CAR-T cells and novel innovative approaches, such as low-intensity pulsed focused ultrasound, aimed at surmounting current roadblocks in GBM CAR-T-cell therapy.

## 1. Introduction

Glioblastoma (GBM) is the most common and aggressive primary malignant brain tumor in adults, with an annual incidence of approximately 3 cases per 100,000 [1,2,3,4].

The current standard of care for GBM includes maximum safe surgical resection followed by radiotherapy with concurrent and adjuvant chemotherapy with or without tumor treating fields [5,6,7]. Despite considerable preclinical and clinical efforts aimed at enhancing therapeutic modalities and extending patient survival, a poor prognosis persists for patients, with a median overall survival ranging between 12 and 18 months post-diagnosis [8]. Therefore, novel, innovative therapeutic approaches are urgently needed.

In recent years, the field of medicine has undergone noteworthy advancements characterized by breakthroughs in the development of innovative therapies designed to strategically modulate the innate capabilities of the immune system for targeting tumor cells [9]. T cells, pivotal in orchestrating adaptive immune responses, have become a central focus in the development of immunotherapy. Advancements in molecular engineering have facilitated the development of new generations of engineered T cells, expressing synthetic receptors known as chimeric antigen receptors (CARs). This innovation enables the targeted and selective elimination of tumor cells in a major histocompatibility complex (MHC)-independent manner, leading to a transformative shift in the treatment landscape for previously untreatable hematologic cancers [10,11,12,13,14]. The promising outcomes witnessed in CAR-T-cell therapy for B-cell malignancies have prompted the commencement of preclinical and clinical studies investigating the application of CAR-T-cell therapy for GBM. In this review, we aim to provide a comprehensive overview of the current challenges and lessons learned from preclinical and clinical studies using CAR-T-cell therapy to treat glioblastoma. Furthermore, we briefly delve into emerging innovative approaches that hold the potential for enhancing the effectiveness of CAR-T-cell therapy in GBM.

## 2. Clinical Trials for CAR-T-Cell Therapy in Adult Patients with GBM

Several tumor antigens have been investigated in small pilot or phase I clinical trials, such as interleukin 13 receptor subunit alpha 2 (IL13Rα2) [15,16,17], epidermal growth factor receptor variant III (EGFRvIII) [18,19], human epidermal growth factor receptor 2 (HER2) [20], erythropoietin-producing hepatocellular carcinoma A2 receptor (EphA2), and disialoganglioside 2 (GD2) [21]. Initial efforts concentrated on IL13Rα2 due to its prevalence in GBMs and its correlation with aggressive tumor behavior and poor prognosis. The inaugural pilot study assessing safety and feasibility, published in 2015, investigated the efficacy of CD8+ CAR-T-cells directed against IL13Rα2 for managing recurrent GBM across three patients. Intracranial administration of CAR-T-cells into the resection cavity demonstrated a satisfactory safety profile for the therapy. While a transient reduction in glioma activity was noted in two patients, examination of tumor tissue from one patient indicated a decrease in overall IL13Rα2 expression [16]. Despite these encouraging findings, all treated patients experienced a recurrence of GBM. In a separate investigation, a patient presenting with recurrent multifocal GBM underwent multiple administrations of CAR-T-cells designed to target IL13Rα2, utilizing two distinct intracranial delivery pathways. This encompassed infusions into the resected tumor cavity followed by infusions into the ventricular system. Notably, no toxicities of Grade 3 or greater were linked to the CAR-T-cell therapy. A favorable clinical response ensued, characterized by regression of all intracranial and spinal tumors, persisting for a duration of 7.5 months subsequent to the commencement of CAR-T-cell therapy [15] (Table 1).

Following these case reports, recently Brown et al. reported the results of the completed NCT02208362 phase I trial assessing the safety and efficacy of CAR-T therapy targeting IL-13Rα2 in 65 patients with recurrent high-grade glioma, primarily recurrent GBM (rGBM) [22]. This study included patients who had undergone extensive prior treatment and did not have specific limitations on factors like tumor size, multifocal disease, prior use of bevacizumab, or the number of prior tumor recurrences. Eligibility for the trial required confirmation of IL-13Rα2 tumor expression, a Karnofsky index of 60 or higher, and a life expectancy of more than four weeks. Fifty-eight patients received at least three CAR-T infusions and were evaluated for disease response (*n*  =  58), overall survival (OS; *n*  =  57), or dose escalation (*n*  =  54). Around 75% of the participants had already experienced their cancer returning at least twice, and most of them had an IDH-wildtype rGBM (41 of 58). The trial investigated three routes of locoregional T-cell administration (intratumoral, intraventricular, and dual intratumoral/intraventricular) and two manufacturing platforms (central memory T cells [Tcm] and naïve, stem cell memory and central memory T cells [Tn/mem]), with the final arm utilizing dual intratumoral/intraventricular delivery and an optimized manufacturing process. In line with findings from the previously mentioned case reports, locoregional CAR-T-cell administration was feasible and well-tolerated, with no dose-limiting toxicities observed across all arms. However, one third of the patients developed Grade 3 toxicity possibly related to CAR-T cells. One patient developed Grade 3 encephalopathy, one patient Grade 3 ataxia, as well as two patients Grade 4 cerebral edema. A clinical maximum feasible dose of 200 million CAR-T cells per infusion cycle was achieved for the optimized arm. Stable disease or better was achieved in 50% of patients, with some partial and complete responses observed. The median overall survival for all patients was 7.7 months, and for the optimized arm, it was 10.2 months. Importantly, three patients who exhibited partial or complete responses had IDH-mutated tumors. Those patients treated with dual (ICT/ICV) CAR-T-cell administration showed the highest INFγ pathway induction in the CSF and slight but statistically significant improvement in overall survival as compared to the other treatment arms.

Moreover, the authors highlighted that the pretreatment of intratumoral CD3 T-cell levels were positively associated with survival. Furthermore, the authors detected the presence of CAR-T cells in the bloodstream already within the first days following CAR-T-cell administration. Notably, their quantity positively correlated with the application of dual delivery, peaking in Arm 5. There was a significant positive relationship between the levels of CAR-T cells in the bloodstream and the expression of CD27 and LAG-3, while conversely, there was a negative correlation with the exhaustion markers PD-1 and CD57. Interestingly, the authors could not find a substantial correlation between CAR-T-cell levels and an escalated dose schedule. These findings suggest that upon introduction into the central nervous system (CNS), these cells can migrate to peripheral regions. This observation carries potential clinical significance and merits further investigation.

Beyond that, a phase I trial examined off-the-shelf, healthy donor-derived, allogeneic steroid-resistant CAR-T cells coupled with recombinant human IL-2 and systemic dexamethasone in a cohort of six patients. The cells were generated from a healthy-donor derived IL13Rα2-targeted CAR+ (IL13-zetakine+) cytolytic T-lymphocyte (CTL) product genetically engineered using zinc finger nucleases (ZFNs) to permanently disrupt the glucocorticoid receptor (GR) (GRm13Z40-2) and endow resistance to glucocorticoid treatment. The treatment was well-tolerated, and transient tumor reduction or tumor necrosis at the site of T-cell infusion was observed in four of the six treated patients. Nevertheless, in this study, all patients suffered from GBM recurrence in the course of the study [17].

EGFRvIII, a mutated variant of the EGFR, represents the predominant form of this receptor in cancer, with approximately half of EGFR-amplified GBM cases expressing it [23]. Despite promising outcomes observed in various preclinical trials indicating a significant reduction in tumor growth, the clinical efficacy of CAR-T-cells specifically targeting EGFRvIII in GBM patients is constrained. O’Rourke et al. investigated in a first-in-human trial intravenous delivery of a single dose of CAR-T cells targeting EGFRvIII in ten patients with rGBM [18]. The median overall survival was approximately eight months, with one patient exhibiting residual stable disease lasting over 18 months. Notably, trafficking of intravenously infused CAR-T-EGFRvIII cells to active GBM regions was observed, accompanied by antigen decrease in five of the seven patients, while no off-tumor toxicity was reported. Following this, Goff et al. combined CAR-T-cell therapy with IL-2 application post-transfer, administered intravenously in 18 patients with rGBM [19]. The median overall survival stood at 6.9 months, with two patients surpassing 1 year and a third patient reaching 59 months. Adverse events included severe hypoxia in two patients, with one resulting in treatment-related mortality, likely due to pulmonary edema resulting from congestion of the pulmonary vasculature caused by activated T-cells. Most patients experienced progressive disease, with a median progression-free survival of 1.3 months.

To mitigate the challenge of recurrent tumor cells expressing wild-type EGFR protein in EGFRvIII CAR-T-cell therapy, Choi et al. investigated in a first-in-human trial open-label study with three rGBM patients using the intraventricular application of an engineered T-cell product (CARv3-TEAM-E) that targets EGFRvIII through a second-generation CAR while also secreting T-cell-engaging antibody molecules (TEAMs) against wild-type EGFR, which is not expressed in the normal brain but is nearly always expressed in glioblastoma [24]. None of the patients developed toxicities over Grade 3; one patient developed transient encephalopathy for 3 days, and one patient fatigue for 8 days. Two patients developed cyclic fevers with transient pulmonary nodules and ground-glass opacities. None of the participants received glucocorticoids during the initial post-treatment phase or for any therapy-related indication. Despite the initial reduction in tumor contrast enhancement, which was consistent with a radiographic response within days of treatment, tumor progression was observed in two of the three participants, which correlated with limited persistence of the CARv3-TEAM-E T cells. Interestingly, CAR-T-cells’ presence was observed in the blood, which peaked 3 weeks after intraventricular administration. At this time, only about 2% or less of the T cells showed surface-bound TEAM while in the CSF samples surface bound TEAM varied between 17.6% and 56.2%, indicating that TEAM-E may facilitate safe and local targeting of wild-type EGFR in the CNS.

Bagley et al. reported preliminary results from a phase I trial involving six patients with rGBM evaluating the safety and determining the maximum tolerated dose of intrathecally administered bivalent CAR-T-cells targeting EGFR and IL13Rα2 [25]. Eligible criteria included isocitrate dehydrogenase (IDH) wild-type GBM after previous radiotherapy and evidence of EGFR amplification confirmed by fluorescence in situ hybridization (FISH) on any previous tumor sample. Important to note is that IL13Rα2 was not mandatory for inclusion. On the other side, patients who received bevacizumab within three months before enrollment or displayed tumors mainly located in the brainstem or spinal cord were excluded from participation. All six reported patients had progressive, multifocal disease at the time of treatment. Neurotoxicity, consistent with immune effector cell-associated neurotoxicity syndrome (ICANS), was observed in all patients at both dose levels tested (10^7^ CAR-T-EGFR-IL13Rα2 cells, *n* = 3 and 2.5 × 10^7^ CAR-T-EGFR-IL13Rα2 cells, *n* = 3), and managed using high-dose dexamethasone and anakinra (IL-1R agonist). One patient at the higher dose level experienced significant toxicity. While early magnetic resonance imaging showed reductions in enhancement and tumor size for all patients and a significant presence of CAR-T-cells and cytokine release was observed in the CSF within the first four days, none met the mRANO response criteria (≥50% decrease in the sum of products of perpendicular diameters of all measurable enhancing lesions sustained for at least 4 weeks).

The HER2 tumor antigen, a receptor tyrosine kinase, is commonly overexpressed in various cancers, including approximately 80% of GBMs. A study was performed to infuse HER2-specific CAR-modified virus-specific T cells in patients with progressive GBM. In this study, a cohort of 17 patients diagnosed with progressive HER2-positive GBM, including 7 individuals under the age of 18, underwent treatment involving one or more infusions of HER2-specific CAR-modified virus-specific T cells, administered without prior lymphodepletion. Notably, these infusions exhibited no significant toxicities. Among the 16 patients evaluated (comprising 9 adults and 7 children), the outcomes varied: 1 patient experienced a partial response lasting more than 9 months, 7 patients maintained stable disease for durations ranging from 8 weeks to 29 months, while 8 patients experienced disease progression. The median overall survival was 11.1 months from initiating the first T-cell infusion and 24.5 months from the initial diagnosis [20].

A recent pioneering clinical trial was conducted involving EphA2-targeting CAR-T-cells intravenously administered to three patients with rGBM. EphA2, a pivotal antigen implicated in tumorigenesis, invasion, and angiogenesis, is expressed in approximately 60% of GBMs. Administered via a single intravenous infusion, the CAR-T-cells triggered pulmonary edema in two patients, a complication that was resolved following dexamethasone treatment. Notably, there were no reported instances of organ toxicity, including neurotoxicity. Outcomes varied, with one patient exhibiting stable disease and two patients experiencing progressive disease, with overall survival durations ranging from 86 to 181 days.

Additionally, one study of GD2-targeting CAR-T cells in a total of eight patients with GBM was investigated. Targeting GD2, which is involved in adhesion and cell signaling, was achieved through intraventricular administration of CAR-T-cell therapy. In addition to intravenous infusion of GD2-specific 4SCAR-T cells, three patients were infused with 4SCAR-T cells into the resected tumor cavity at the same time. The therapy was well-tolerated, with no severe adverse events reported. While some patients experienced a longer lifespan and specific loss of GD2 antigen, the study’s small sample size prevented a clear determination of clinical benefits [21].

Beyond that, B7-H3, a type I transmembrane protein active in immune checkpoint regulation, has been shown to have elevated expression in high-grade gliomas, with increased expression negatively correlated with survival prognosis [26]. Preclinical studies have displayed effective targeting and tumor regression [27]. Tang et al. reported the first patient with rGBM treated with intraventricular infusion of B7-H3 CAR-T-cells and reported a transient reduction in tumor enhancement which was persistent for 50 days. The patient was treated with a total 79 million CAR-T-cells over seven cycles but eventually recurred with a new onset of neurological deficits [28].

In summary, most clinical trials have observed a transient anti-glioma response with manageable side effects, providing hope for future CAR-T-cell immunotherapy endeavors targeting glioma. However, while CAR-T-cell therapy holds promise for treating GBM, the trials did not yield significant success. CAR-T has to face several challenges such as the limited accumulation and persistence of T cells in the CNS, T cell exhaustion, and antigen loss, as well as local and systemic immunosuppression. Subsequent sections will delve into the current challenges of CAR-T-cell therapy for glioma and explore potential solutions.

**Table 1:** Summary of CAR-T-cell clinical trials in glioblastoma.

Author/ Ref	Year	Target Antigen	Conditions	NCT No	Phase	Number of Patients	Lessons Learned
Brown et al. [16]	2015	IL13Rα2	Recurrent or refractory unifocal supratentorial WHO Grade 3 or 4 glioma	NCT00730613	N/A (pilot study)	3	Intracranial delivery of CAR-T cells via reservoir/catheter system allows repetitive dosing, no adverse therapy-related side effects, and transient anti-glioma activity
Brown et al. [15,22]	2016 2024	IL13Rα2	Recurrent multifocal GBM	NCT02208362	Phase I	65 (reported)	2016: Case Report: CNS tumors including spinal tumors regress in intraventricular administration of CAR-T cells, absence of systemic toxicity, consistent effect for 7.5 months 2024: Stable disease or better in 50% of patients, with two partial responses, one complete response, and a second complete response after additional CAR-T cycles off protocol, for recurrent GBM combined intraventricular and intratumoral application of CAR-T showed the best survival (7.7 months vs. 10.2 months)
Brown et al. [17]	2022	IL13Rα2	Progressive or recurrent WHO Grade 3 or 4 malignant glioma	NCT01082926	Phase I	6	No graft-versus-host disease, no device-related adverse effects, and evidence of local tumor necrosis
O’Rourke et al. [18]	2017	EGFRvIII	Recurrent GBM	NCT02209376	Phase I	11	No therapy-related toxicity or cytokine release syndrome, trafficking of CAR-T cells to the tumor site, EGFRvIII antigen loss
Goff et al. [19]	2019	EGFRvIII	Recurrent GBM	NCT01454596	Phase I	18	No dose-limiting toxicities until the highest dose (≥10^10^) No objective responses observed
Choi et al. [24]	2024	EGFR and EGFRvIII	Recurrent GBM	NCT05660369	Phase I/pilot	3	CARv3-TEAM-E T cells, designed to target EGFR variant III and wild-type EGFR, showed promising safety profiles without severe adverse events or dose-limiting toxic effects, transient tumor regression
Bagley et al. [25]	2024	EGFR and IL13Rα2	Recurrent GBM	NCT05168423	Phase I	6	Preliminary Case Series: Intrathecally delivered bivalent chimeric antigen receptor (CAR) T cells targeting EGFR and IL13Rα2 induced early-onset neurotoxicity which was effectively managed using high-dose dexamethasone and anti-IL1R treatment), none of the patients met the criteria for an objective response rate
Ahmed et al. [20]	2017	HER2	Progressive recurrent GBM (WHO Grade 4 glioma)	NCT01109095	Phase I	16	1 × 10^6^/m^2^–1 × 10^8^/m^2^ T cells were infused without severe adverse effects, 50% of the patients had objective responses
Lin et al. [29]	2021	EphA2	Recurrent GBM	NCT03423992	Phase I	100	1 × 10^6^ cells/kg were infused and well-tolerated and transient efficacy in three patients was observed
Tang et al. [28]	2021	B7-H3	Recurrent GBM	-	-	1	Case Report: Intraventricularly administered T cells mediated short-term anti-tumor response
Liu et al. [21]	2023	GD2	Recurrent or progressive GBM	NCT03170141	Phase I	20	Single and combined infusions of CAR-T cells were safe and well-tolerated Partial antigen loss

## 3. Immune Privilege of the CNS Parenchyma

**Figure 1:**
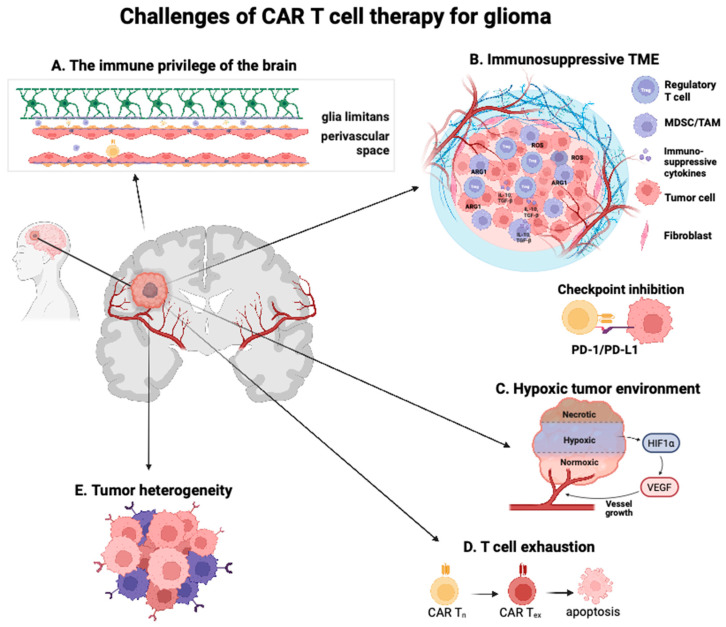
Challenges of CAR-T-cell therapy for glioma. (**A**). Immune privilege of the brain poses physical and cellular barriers to CAR-T-cell homing. One of these barriers is the blood–brain barrier, which is composed of the endothelial cells of the capillary wall, astrocyte end-feet sheathing the capillary, and pericytes embedded in the capillary basement membrane. At the level of the postcapillary venules, the adjacent basement membranes leave a virtual perivascular space in which occasional APCs are embedded that play an important role in reactivating T cells, enabling them to cross the glia limitans. (**B**). The immunosuppressive TME comprises pro-tumoral myeloid cells, immunosuppressive cytokines/chemokines, and checkpoint molecules, all of which contribute to inhibiting CAR-T-cell efficacy and activation. (**C**). Hypoxia in GBM creates an inhospitable environment that severely limits oxygen and nutrients to CAR-T cells. (**D**). Chronic stimulation and exposure to antigen results in CAR-T-cell exhaustion. (**E**). Inter and intra-tumoral heterogeneity pose a significant impediment to designing CAR constructs and choosing target antigens.

A significant impediment to the efficacy of immunotherapy for glioma lies in the immunological privilege of the CNS (Figure 1A). Over a century ago, experimental evidence substantiated the immunological privilege of the CNS parenchyma in comparison to the remainder of the body, owing to anatomical impediments that restrict the ingress and egress of immune cells and CNS antigens [30]. Consequently, CNS antigens and brain neoplasm frequently elude detection by the peripheral immune system. The CNS parenchyma’s status as an immune-privileged organ derives from its ability to tolerate experimentally grafted tissue without an immune response, resulting in tissue rejection [31,32]. Historically, immune privilege has been demonstrated as early as Shirai’s 1921 experiments, where rat sarcoma grew successfully without immune rejection following direct implantation into the brain parenchyma yet was rejected upon subcutaneous or intramuscular injection [33]. Findings by Murphy and Sturm in 1923 demonstrated that immune reactions can occur within the CNS via direct communication with the host immune system, as sarcoma transplants experience rejection if co-transplanted with recipient spleen tissue [34]. Additionally, Murphy and Sturm observed that transplanted sarcoma when located near CSF-containing ventricles was rejected. Hence, this underscored that the immune privilege is confined to the brain parenchyma, a phenomenon often referred to as the ‘compartmentalization of immune privilege’ [32].

Modern imaging and cellular profiling technologies have facilitated a more comprehensive characterization of distinct compartments. Recent findings have substantiated the presence of a dural lymphatic system and delineated cerebrospinal fluid (CSF) drainage pathways, such as those traversing the cribriform plate towards the nasal mucosa, ultimately draining into the deep cervical lymph nodes [35,36]. In contrast to the CSF space, the CNS parenchyma lacks a specialized lymphatic drainage system. The glymphatic system, a recently identified mechanism proposing an interstitial fluid (ISF)–CSF exchange system, suggests that CSF enters the brain through arterial perivascular vessels, engages in exchange with ISF through aquaporin 4 (AQP4) water channels, and subsequently exits into venous perivascular vessels, draining into the deep cervical and lumbar lymph nodes [37]. However, the efficiency of antigen drainage via this system into the periphery remains a subject of ongoing investigation.

One of the inherent obstacles that immune cells need to overcome when reaching the CNS parenchyma is the blood–brain barrier (BBB), consisting of capillary endothelial cells, their basement membrane, the perivascular space, and the glia limitans. The glia limitans serves as a concluding barrier created by the projections of astrocytes, representing a hurdle that immune cells must traverse to move from the cerebrospinal fluid (CSF)-filled perivascular space into the parenchyma [38]. GBM is characterized by areas with different BBB integrity, with the tumor mass consisting of areas with highly dysregulated and dysfunctional BBB due to aberrant vasculature in areas of contrast enhancement within the tumor but a relatively intact BBB at the non-enhancing tumor edges [39].

Immune cells, such as CAR-T-cells, have to undergo a multistep process to home into the CNS parenchyma. Firstly, circulating T cells are tethered, rolling across the endothelium in a process driven by calcium-binding interactions between endothelial selectins and carbohydrates expressed on the T cell surface [40,41]. A reduction in T cell circulation speed allows for the stimulation of T cell G-protein coupled receptors by chemokines present on the endothelial cell surface [42]. GPCR activation increases the affinity of T cell surface integrins for endothelial surface intracellular adhesion molecule-1 (ICAM-1), with binding resulting in the arrest and flattening of the T cell on the endothelial cell surface [40,43]. Lastly, T cells migrate out of the postcapillary venules via paracellular diapedesis, in which adhesion interactions drive the T cell through the tight junctions between endothelial cells [44]. It is important to note that the activation of T cells is key for the passage through the vascular endothelium. Preclinical BBB modeling has indicated that GBM-targeting activated CAR-T cells show excellent homing via the BBB [45]. Furthermore, in the context of CD19 CAR-T-cell therapy, endothelial cell activation and BBB disruption have been observed after adoptive T cell transfer [46]. Passage through the vascular endothelium is followed by T cell migration through the glia limitans, a process initiated by T cell reactivation through recognition of its cognate antigen on perivascular or leptomeningeal antigen-presenting cells (APCs) [30,47]. To our knowledge, studies specifically investigating the interaction of CAR-T cells with perivascular APCs in the perivascular space have not yet been conducted. However, we assume that disruption of the glia limitans occurs in a similar manner, as it has been observed with conventional T cells via the production of matrix metalloproteinases (MMP) 9 and 2 by recruited myeloid cells resulting in the selective cleavage of dystroglycan on the astrocyte foot-process [40,48,49].

### Alternative Delivery Routes

As the efficient homing of CAR-T-cells into the brain determines the efficacy of anti-tumor immunity, novel approaches to enhance the presence of CAR-T-cells are urgently needed. Locoregional delivery modalities, such as intraventricular (ICV), intracavitary (IC), or intratumor (IT) immunotherapy applications, have been successfully investigated in multiple human phase I GBM trials as an alternative to surmounting inherent biological barriers [15,18,22,50]. These targeted delivery routes hold the potential to achieve lower drug concentrations, thereby mitigating the risk of off-target toxicities. However, recent clinical trials have reported the presence of ICV/IT-administered CAR-T-cells in the periphery, and further investigations are needed to determine if local delivery can truly prevent off-target toxicities [22]. Furthermore, all techniques require invasive surgery. In this context, low-intensity pulsed focused ultrasound, combined with microbubble application (LIPU+MB), provides a means to temporarily breach the BBB. This allows for the transient influx of immune cells or facilitates the transport of drugs into the brain. Focused ultrasound implements a concave transducer to convert sound waves into a focused beam. It is used in tandem with intravenously administered microbubbles ranging from approximately 1–5 μm in diameter, consisting of lipid-encapsulated gases, such as perfluorocarbon or sulfur hexafluoride [51,52,53]. The ultrasonic wave emitted by the transducer interacts with the microbubble-encapsulated gas via cavitation, a process consisting of expansion and contraction of the microbubbles in response to a specific excitation frequency generated by compression and refraction of the ultrasonic pressure wave. In stable cavitation, low-intensity pulsed ultrasound (LIPU) causes harmonic oscillatory expansion and contraction of the microbubbles. This steady, vibratory motion initiates a process known as microstreaming, where the fluid surrounding the microbubbles begins to flow and exert mechanical force on the endothelium, resulting in temporary rupture of the bonds between tight junction proteins in the BBB [51,54,55].

Initial preclinical and clinical studies in the field of malignant gliomas have shown improved infiltration of immune cells into the tumor, and recently Sonabend et al. demonstrated in a pioneering phase I trial where patients received a skull-implantable ultrasound device that repeated sonication treatment is safe and allows a repeated increase in delivery of albumin-bound paclitaxel and carboplatin chemotherapies into the brain [56,57,58]. Moreover, the immunomodulatory effects of LIPU+MB have been considered in the context of inducing transient inflammation, which may subsequently enhance the favorable recruitment of immune cells into the CNS [56,59,60]. However, comprehensive clinical studies are imperative to better understand the mechanisms by which LIPU+MB can facilitate the homing of CAR-T cells in human GBM.

## 4. Tumor Microenvironment

Once CAR-T-cells reach the TME, they have to face many challenges, including (1) direct suppression by tumor cells and tumor-infiltrating immune cells and (2) depletion of environmental nutrients, including oxygen, amino acids, and others. These challenges result in T cell exhaustion, reduced killing functionality, and impaired persistence and survival. Here, we outline preclinical strategies for improving overall CAR-T-cell function in the TME.

### 4.1. Local and Systemic Immunosuppression

The TME of GBM is considered immunologically ‘cold’, largely due to its poor effector T cell infiltration [61]. GBM tumors are heavily infiltrated with suppressive myeloid cells [62,63], regulatory T cells (Tregs), and stromal cells that produce high levels of immunosuppressive cytokines. Myeloid-derived suppressor cells (MDSCs) and tumor-associated macrophages (TAMs) can comprise over 30% of the total tumor mass, and infiltration of these cells is associated with a worse prognosis [64]. Tumor-infiltrating MDSCs suppress cytotoxic T cell activity by inducing oxidative stress and secrete reactive oxygen species (ROS) [65,66]. Indoleamine 2,3-dioxygenase 1 (IDO), Arginase (ARG1), and inducible nitric oxide synthase (NOS2) are all utilized by MDSCs to alter the TME [67,68,69] (Figure 1B). IDO depletes tryptophan, ARG1 depletes arginine, and all three enzymes enrich reactive nitrogen species, which inhibits CD3ζ chain expression and induces T cell apoptosis [70,71,72]. Tumor-infiltrating TAMs have an increased expression of immunosuppressive markers, including fms-like tyrosine kinase 3 (FLT3) and transforming growth factor beta (TGFβ) [73]. Further, tumor cells secrete TGFβ, inhibiting T cell effector function and promoting Tregs. Tregs are immunosuppressive T cells that negatively modulate the immune response and, therefore, prevent autoimmunity. They are actively recruited by GBM tumor cells and constitute up to 30% of infiltrating lymphocytes in GBM [74].

Moreover, GBM cells and TAMs produce molecules known as immune checkpoint ligands, including programmed cell death ligand 1 (PD-L1). These ligands inhibit anti-tumor effector cells via programmed cell death protein 1 (PD-1) and cytotoxic T-lymphocyte-associated protein 4 (CTLA-4). The latter binds CD80 and CD86 expressed by APCs at high-affinity levels. Consequently, CD28, a co-stimulatory receptor present on T cells, cannot bind to its ligands, thereby impeding the activation process of T cells [75,76].

Furthermore, recent analyses of clinical samples from GBM patients have uncovered that, beyond localized immunosuppressive effects, there is a pronounced systemic immunosuppression and lymphopenia observed in these individuals [77,78]. Notably, CD4 T-cell levels in GBM patients approach the nadir observed in individuals with acquired immune deficiency syndrome (AIDS), underscoring the severity of the immune suppression. Despite its critical importance, research into systemic immunosuppression in GBM is sparse, with only a limited number of studies exploring the intricate network and mechanisms underpinning this phenomenon.

Investigations have revealed that GBM may induce the sequestration of naïve T cells within the bone marrow, a process potentially exacerbated by the loss of sphingosine-1-phosphate receptor 1, which is crucial for the systemic circulation of T cells [77]. Furthermore, systemic immunosuppression has been attributed to a potent, non-steroidal serum factor that impedes T cell proliferation, suggesting the involvement of multiple elements in the immunosuppressive landscape of GBM.

The epidemiological characteristics and clinical management of GBM further contribute to systemic immunosuppression. Primarily, the median age of GBM diagnosis is 64 years, predominantly affecting older individuals who naturally experience age-related declines in immune function [79]. This demographic is characterized by a reduced capacity for T cell production due to diminished bone marrow and thymic activity. Additionally, the standard-of-care treatments for GBM, including surgical interventions, chemotherapy, and radiation therapy, can induce iatrogenic immunosuppression, leading to treatment-related lymphopenia [80]. Moreover, the widespread use of dexamethasone for managing brain edema in GBM patients not only reduces populations of naïve and memory T cells but also promotes the proliferation of immunosuppressive myeloid cells [81].

In the following paragraphs, we will explore potential strategies to mitigate or reverse the immunosuppressive TME.

#### 4.1.1. TAM-Targeted CAR-T-Cells

The importance of addressing immunosuppressive myeloid cells is evident in two recent studies by Rodriguez-Garcia et al. and Sanchez-Paulete et al. [82,83]. In these studies, the authors describe the use of CAR-T-cells to target and eliminate TAMs. Sanchez-Paulete et al. use a broad pan-macrophage approach with anti-F4/80 CARs, while Rodriguez-Garcia et al. describe a CAR targeting folate receptor β (FRβ), which is highly expressed on M2-like tumor supporting macrophages. Interestingly, the broad elimination of tumor macrophages with an anti-F4/80 CAR led to delayed tumor growth and resulted in CAR-derived-IFNg expression, which resulted in immune editing of the tumor in lung, ovarian, and pancreatic cancer models. M2 macrophage elimination by FRβ CAR-T cells resulted in enrichment of CD8 T cells in vivo and resulted in a survival benefit in mouse models. Interestingly, the most potent anti-tumor effect was seen when animals were pretreated with FRβ CAR-T cells, followed by tumor-directed anti-mesothelin CAR-T cells. These creative approaches involving TME conditioning highlight the many applications for CAR-T cells that extend beyond targeting tumor cell killing.

#### 4.1.2. Targeting TGFβ

Tumor cells often employ TGFβ secretion as a mechanism to evade the immune system, facilitating tumor progression. TGFβ plays a pivotal role in various cellular processes, including cell growth, proliferation, differentiation, apoptosis, angiogenesis, and cellular homeostasis. Within the immune system, TGF-β acts to suppress effector responses in APCs, memory T cells, and effector T cells. Upon secretion, soluble TGFβ dimers engage with TGFβ receptor II (TβRII), leading to the recruitment of TGFβ receptor I (TβRI) and the formation of a tetrameric complex comprising two of each receptor. Subsequent phosphorylation of TβRII activates TβRI, initiating a signaling cascade characterized by the phosphorylation of SMAD protein complexes. Phosphorylated SMAD2/3 (p-SMAD2/3), and to a lesser extent p-SMAD1/5/8, translocate to the nucleus, where they bind to DNA, promoting the expression of genes regulated by TGF-β. This signaling pathway typically induces growth arrest and apoptosis in cancer cells. However, cancer cells may evade these effects through mutations in their TGFβ receptor or SMAD protein genes. To mitigate the inhibitory effects of TGFβ on tumor infiltrating lymphocytes various strategies have been explored to render these cells less responsive to TGFβ [84,85,86]. TGFβ-mediated immunosuppression has been targeted by various approaches, including anti-TGFβ monoclonal antibodies and transducing T cells with dominant negative TGFβ receptors, making them unresponsive to TGFβ signaling [87,88]. A 2018 study by Hou et al. explored the use of TGFβ-responsive CAR-T cells [89,90]. There, CAR-T cells promote anti-tumor immunity by secreting Th1 cytokines in response to TGFβ signaling and reducing the impact of TGFβ on surrounding immune cells.

#### 4.1.3. Checkpoint Inhibition

Immune checkpoint inhibitors block inhibitory pathways, which helps immune evasion of GBM [91]. In GBM, tumor cells and immune cells, such as CD4+, CD8+ T cells, and TAMs, express programmed death ligand 1 (PD-L1) [91,92]. In patients with GBM, there is a positive correlation between PD-L1 and glioma grade and low survival rate [92]. Our study showed that a combination of radiotherapy and antibodies inhibiting PD-L1 increased survival in murine glioma model [93]. Another study of a mouse glioma model showed that PD-L1 expression is decreased by inhibiting IL-6, which led to an increased survival rate and decreased tumor growth in mice [94]. In GBM, PD-L1 was expressed in 88% of newly diagnosed patients and 72.2% of recurrent patients [95]. Another study found that 61% of GBM patients have tumor cells that express PD-L1 among 94 patients [96].

Another target for checkpoint inhibition is T-cell immunoglobulin domain and mucin domain protein 3 (TIM3). In GBM, TIM3 is expressed in both tumor and immune cells and is highly upregulated compared to other checkpoint molecules [97,98,99]. Like other checkpoint molecules, TIM3 is involved in immune tolerance and is able to bind to multiple targets, including cancer-embryonic antigen cell adhesion molecule 1 (CEACAM), galectin-9 (Gal-9), high mobility group protein 1 (HMGB-1), and phosphatidylserine (PtdSer), leading to T cell apoptosis or inhibition. Further, interaction between TIM3 and IL-6 results in macrophage recruitment and polarization to an M2 phenotype [100]. In mouse models of GBM, anti-TIM-3 and anti-CEACAM blockades resulted in improved survival [101]. A currently ongoing phase I clinical trial (NCT03961971) is investigating anti-TIM-3, anti-PD-1, and stereotactic radiotherapy in recurrent GBM patients [102].

Cytotoxic T-lymphocyte-associated protein 4 (CTLA-4) is a checkpoint molecule that is expressed on T cells after stimulation. CTLA-4 competes with CD28 in binding CD80/CD86, which can result in T cell inactivation [103]. Various clinical trials have explored the use of anti-CTLA-4 monoclonal antibodies in conjunction with anti-PD-1 antibodies in recurrent GBM (NCT03233152, NCT02794883) [104]. Interestingly, a 2023 study from Agarwal et al. investigated the deletion of CTLA-4 in CAR-T cells in leukemia and lymphoma models [105]. The deletion of CTLA-4 resulted in robust CD28 signaling in CAR-T-cells, improved expression of the CAR construct, and improved killing efficacy. Importantly, CTLA-4 deletion allowed for the activation and killing of dysfunctional patient-derived CAR-T cells that had previously failed in clinical settings. This study highlights the importance of checkpoint molecules, not only as targets for checkpoint inhibition but also as considerations when designing novel CAR constructs.

Thus far, clinical trials implementing checkpoint inhibition therapy for GBM have demonstrated limited efficacy, which may be attributed to the inadequacy of checkpoint inhibition as a standalone intervention [106,107,108,109]. Nevertheless, a combination of checkpoint inhibition with CAR-T-cell therapy has the potential to amplify the effectiveness of CAR-T-cells, a concept supported by preclinical studies [110]. For example, inhibiting immune checkpoints such as PD-1, CTLA-4, and TIM-3, alongside CAR-T-cell therapy, have demonstrated increased treatment efficacy in a D270 mouse model, evidenced by better control of tumor growth and extended survival. The extent of the improvement provided by checkpoint inhibitors appears to be contingent upon the specific CAR-T-cell utilized, as CAR-T cells directed against EGFRvIII and IL13Rα2 each promoted distinct immune checkpoint environments within their respective tumor settings [18]. Clinical trials are now underway to evaluate the safety and efficacy of this combined approach in GBM patients, including trials using CAR-cells against EGFRvIII with Pembrolizumab (NCT03726515) and CAR-T-cells aimed at IL13Rα2 in conjunction with Nivolumab or Ipilimumab (NCT04003649).

### 4.2. Hypoxia and Metabolism

GBM tumors have highly hypoxic and necrotic regions [111,112]. Compared to a healthy brain, the tumor vasculature is often tortuous and dysfunctional, with heterogeneous vascular permeability [113]. This results in decreased oxygen and nutrient availability in the TME [114]. The master transcription factor hypoxia-inducible factor 1a (HIF-1a) is stable under hypoxic conditions but is rapidly degraded in the presence of oxygen. In the context of GBM, HIF-1a plays a role in regulating tumor metabolism and increases GBM aggressiveness by promoting angiogenesis via upregulation of vascular endothelial growth factor (VEGF), erythropoietin (EPO), and platelet-derived growth factor (PDGF) family proteins [115,116,117] (Figure 1C). HIF-1a-mediated lactate accumulation via the glycolytic pathway promotes tumor growth and reduces NK and T cell effector functions [118,119]. Further, HIF-1a can act on over 100 transcriptional targets to activate autophagy pathways and support an immunosuppressive TME [114]. In the tumor immune microenvironment, HIF-1 signaling induces the expression of prostaglandin E2 (PGE2) and PD-1/PD-L1 [120,121,122]. Further, HIF-1 contributes to tumor immune evasion by blocking the presentation of tumor antigens on MHC I and downregulating NKG2D, decreasing NK cell toxicity [123].

The impacts of hypoxia in the nutrient-poor tumor microenvironment can also be an impediment to CAR-T-cell therapy, decreasing the efficacy and fitness of these cells [124]. To effectively control a tumor, CAR-T cells must be activated, where they undergo expansion and differentiation, eventually leading to the cytolysis of tumor cells. These are high-energy functions that require a rich supply of oxygen and nutrients, a reality incompatible with the GBM microenvironment [125]. Further, the underdeveloped vasculature of GBM can hinder CAR-T-cell extravasation into tumor tissue by downregulating vascular cell adhesion molecule 1 (VCAM1) [126].

Another abnormal metabolic characteristic of GBM is the depletion of certain amino acids from the TME, primarily tryptophan, arginine, and glutamine [127,128]. Catabolism of these amino acids partially drives the immunosuppression environment of GBM, as T cells rely on these for activation, proliferation, and cytolysis [129]. As mentioned above, immunosuppressive myeloid cells are widely known to express the enzymes IDO and ARG1, which deplete tryptophan and arginine, respectively [130]. CAR-T-cell therapy paired with IDO and arginase inhibitors have been shown to enhance interferon-γ (IFNγ) secretion and proliferation [131].

Re-wiring T cell metabolism to carefully regulate the balance between glycolysis and oxidative phosphorylation (OXPHOS) may be a promising tool to overcome the tumor’s metabolically abnormal environment. Effector T cells primarily use glycolysis, while memory T cells rely heavily on OXPHOS [132]. Depending on the desired outcome, the knockout or overexpression of certain metabolic proteins can favor and reduce mitochondrial bioenergetics [133]. The knockout of AMP-dependent kinase a1 (AMPKa1) results in a robust antitumor response (reduced mitochondrial bioenergetics), while the overexpression of peroxisome proliferator-activated receptor gamma coactivator 1a (PGC1a) leads to a robust memory and re-challenge response (enhanced mitochondrial bioenergetics) [134,135].

A 2023 study from our group aimed to mitigate hypoxia-induced CAR-T-cell inhibition by pretreating cells with metformin, an AMPK activator, and rapamycin, a mammalian target of rapamycin (mTOR) inhibitor [112]. Pretreated CAR-T cells were shown to have activated PPAR-gamma coactivator 1a (PGC-1a), resulting in enhanced mitochondrial respiratory capacity. Ultimately, Met-Rap treatment of CAR-T cells demonstrated robust persistence and glioma tumor cell killing in hypoxic conditions and extended survival in mice with orthotopic SB28-EGFRvIII tumors. Further, the Met-Rap treated mice had fewer tumor-infiltrating monocytic MDSCs than control mice [112].

#### 4.2.1. Hypoxia-Sensing CARs

In efforts to reduce off-tumor toxicity and exploit intrinsic characteristics of tumors, hypoxia-sensing CARs have been developed. Interestingly, unlike previous CARs that rely on intra-tumoral antigen, hypoxia-sensing CARs use physical cues for promoting activation. Studies from Kosti et al. (2021) and Zhu et al. (2023) describe hypoxia-sensing and hypoxia-responsive next-generation CAR-T cells in various solid cancers [136,137,138]. Kosti et al. explore the development of an ErbB CAR, in which expression of the CAR construct is controlled by a hypoxia-sensing switch, and oxygen saturation is inversely correlated with CAR expression [136]. This allows for less on-target, off-tumor killing, as the CAR is expressed only in the hypoxic tumor environment. In this system, termed “HypoxiaCAR,” the CAR construct contains both an oxygen-dependent degradation domain and nine hypoxia-responsive elements (HRE). This results in CAR expression that is mediated by HIF1a. This dual system resulted in less leaky CAR expression and strong CAR expression under 0.1% O_2_ hypoxic conditions. Importantly, the HypoxiCAR was demonstrated to be reversible, and CAR expression could be toggled on or off depending on oxygen availability. Zhu et al. report a similar approach (5H1P-CEA CAR-T), which uses five tandem VEGF HRE repeats linked to a cytomegalovirus (CMV) minimal promoter [137]. In vitro, their inducible CAR-T cells exhibited low cytokine release and cytotoxicity under normoxic conditions, but efficiently killed tumor cells under hypoxic conditions compared to controls. Further, 5H1P-CEA CAR-T cells adopted a central memory or effector memory phenotype, while controls had a greater proportion of naïve T cells. Finally, Zhu et al. showed that hypoxia-inducible CAR expression results in better oxidative metabolism and decreased T cell exhaustion in vivo.

#### 4.2.2. MEK Inhibitors

Under conditions of persistent stimulation in the TME, T cells may excessively proliferate, resulting in diminished effector function. To mitigate this, approaches targeting the mitogen-activated protein kinase (MAPK) pathway, specifically mitogen-activated protein kinase kinase 1/2 (MEK1/2) have emerged. MEK1/2 inhibitors in T cells function by metabolic modulation and delaying cell cycle progression by cyclin D1 suppression [139]. In a 2021 study, Verma et al. show that MEK1/2 inhibition results in fatty acid oxidation-driven mitochondrial biogenesis and adoption of a stem cell-like memory phenotype [139,140]. T cells treated with MEK inhibitors had a robust killing ability, proliferative capacity, and plasticity, suggesting that this strategy may aid in the robustness and persistence of CAR-T cells in vivo.

## 5. Mitigating T Cell Exhaustion

To mount an effective anti-tumor response, CAR-T-cells must exhibit optimal fitness to prevent T-cell exhaustion (Figure 1D). In this context, continuous activation can lead to T cell exhaustion; however, conversely, some level of tonic signaling has been hypothesized to potentially benefit CAR-T-cells maintaining them to be more alert to potential antigens. In the following section, we will briefly discuss how CAR design can impact T cell fitness.

### 5.1. CAR Construct Modification

The structure of CARs consists of four main components: the extracellular antigen-binding region, hinge region, transmembrane region, and intracellular signaling region [141]. The antigen binding domain is a single chain variable fragment (scFv) comprising a variable heavy chain and light chain of monoclonal antibodies connected with a linker [141]. scFv is essential for the affinity and specificity of CARs in binding to the target epitopes [142]. The hinge region is an extracellular structure, providing flexibility to surmount steric hindrance, allowing the antigen binding domain to access the targeted epitope [141]. The transmembrane region is mainly known for anchoring the CAR to the T-cell membrane [141], although other studies suggest that it has effects on CAR expression, signaling, stability, and dimerization with signaling molecules [143,144]. First-generation CARs have only a CD3ζ or FcRγ intracellular domain [145]. Second-generation CARs are characterized by incorporating one costimulatory domain, such as one derived from CD28 or CD137, in conjunction with a CD3ζ intracellular domain. This design serves to improve the signaling strength and potency of CAR-T-cells [146,147,148]. To further enhance signaling capabilities and functional efficacy, third-generation CARs have two costimulatory CD3ζ intracellular domains [149]. The type of costimulatory domain included can induce differential gene expression, influences T cell subset formation, functionality, and exhaustion. Current CAR construct designs employ a combination of costimulatory domains from CD28, CD27, OX40, or 4-1BB, or implement an inducible costimulatory domain (ICOS) to regulate T cell signaling and subsequent downstream pathway activation [150]. Constructs incorporating a CD28-CAR domain display faster proliferation and activation and an increased rate of glycolysis, yet display lowered T cell persistence [144,151]. CARs containing the CD28 domain can be come rapidly exhausted due to the clustering of the CAR single-chain variable fragment. This induces CAR-CD3ζ tonic signaling and accumulation of NFAT transcription factors upon CD28 costimulatory domain activation [151]. In contrast, 4-1BB and ICOS incorporating CARs display a longer duration of functionality and increased levels of oxidative phosphorylation, at the expense of lowered potency [144,151]. Studies have shown that 4-1BB costimulatory domain CAR-T- cells display elevated levels of memory T cell markers, which may aid in mitigating CAR-T-cell exhaustion [152,153].

### 5.2. TRUCKs

The fourth generation of CAR-T cells incorporate signaling domains derived from cytokine receptors or possess the capacity for inducible expression of inflammatory cytokines such as IL-7, IL-12, IL-18, IL-23, and others [154,155,156,157]. Termed “T-cells redirected for universal cytokine-mediated killing (TRUCKs),” these advanced CAR-T-cells induce local nuclear factor of activated T cells (NFAT)-dependent cytokine expression after cognate tumor antigen binding [158]. Specifically, CAR signaling leads to NFAT phosphorylation that ultimately drives the expression of a transgenic protein, a cytokine. This strategy allows for both simultaneous CAR-T-cell-mediated killing and immune modulation of the TME via secretion cytokines, which can elicit both autocrine (IL-7, IL-15, IL-8) and paracrine (IL-12) downstream effects and can (1) promote the survival of CAR-T cells and (2) condition and modulate the TME by repolarizing TAMs or activating NK cells [155,159,160]. Upon activation, the expression of autocrine-acting cytokines may continue to sustain survival of the CAR-T-cell, improving persistence in vivo. Further, inducible expression of cytokines improves safety by limiting the toxicity associated with system administration of cytokines, such as IL-12 [161]. Multiple phase I clinical trials (NCT03542799, NCT03932565) have reported the use of TRUCKs for recurrent ovarian cancer (MUC16^ecto^ CAR + IL-12) [156], Nectin4-expressing solid tumors (Nectin4/FAP CAR + IL-7 and CCL19 or IL-12), and metastatic colon cancer (EGFR CAR +IL-12).

### 5.3. SynNotch CAR-T-Cells

Recent discoveries have highlighted the utility of chimeric forms of Notch, a type-1 transmembrane protein, wherein both the extracellular sensor module and the intracellular transcriptional module are substituted with heterologous protein domains. These chimeric Notch constructs, termed synthetic Notch (synNotch), provide a versatile platform for creating novel cell–cell contact signaling pathways, such as the induction of CARs (SynNotch CAR) [162,163,164].

Choe et al. (2021) have leveraged the SynNotch CAR system to engineer innovative T cell circuits based on a “prime-and-kill” strategy [165]. In this approach, the initial antigen, exclusively expressed on brain or GBM cells, primes the T cells to induce the expression of a CAR-targeting antigens such as IL-13Rα2 and EphA2, thus eliminating GBM cells expressing either EphA2 or IL-13Rα2. Importantly, the SynNotch system mitigates tonic signaling and reduces T cell exhaustion. Traditional CAR-T-cells constitutively express the CAR construct, resulting in a greater probability for exhaustion and decreased persistence. Compared to constitutively expressing CAR constructs, SynNotch CARs have a more predominately naïve/stem cell memory phenotype. Further, SynNotch CAR-T-cells have a lower expression of the checkpoint molecules PD-1, LAG3, and TIM3, suggesting that these have a less exhausted phenotype [165].

The EGFRvIII–SynNotch primed EphA2/IL-13Rα2 CAR (E-SYNC) system is selectively activated by EGFRvIII as the GBM-specific signal, leading to the complete eradication of patient-derived xenografts (PDX) with heterogeneous EGFRvIII expression. Importantly, this approach spares EphA2/IL-13Rα2-positive cells outside of the CNS and a significant proportion of T cells engineered with SynNotch CAR circuits exhibit a stem/naïve cell state, associated with remarkable in vivo persistence [165]. These findings instill optimism for the auspicious application of SynNotch CAR-T cells in GBM, further underscored by the recent approval from the United States FDA to commence the inaugural human trial.

### 5.4. SNIPRs

Synthetic intramembrane proteolysis receptors (SNIPRs) consist of a humanized SynNotch receptor capable of tunable extracellular sensing and intracellular transcriptional response capabilities [166,167]. Due to their incorporation of fully humanized components, SNIPRs minimize the risk of immune rejection and are compatible with human transcription factors and humanized synthetic transcription factors. SNIPRs are notable for enhanced customizability over the SynNotch circuit due to the ability of the transmembrane (TMD) and juxtamembrane (JMD) domains to be modified through amino acid substitution or deletion. Modification of the TMD and JMD domains allows for fine-tuning of the SNIPR sensitivity, preventing T cell background signaling activity, and for a reduction in the overall size of the receptor [167]. Importantly, the SNIPR system results in less T cell exhaustion by preventing T cell background signaling. Unlike conventional CARs, which have a baseline level of tonic signaling, SNIPRs express the CAR construct at tumor sites only, greatly reducing tonic signaling and combatting the major impediment of T cell exhaustion [168]. The integration of SNIPRs into current SynNotch receptor constructs represents a promising optimization of current SynNotch designs targeting glioblastoma due to the enhanced customizability afforded by the TMD and JMD when paired with the extracellular recognition domain and intracellular response domain employed by the SynNotch circuit [165,167].

### 5.5. Gene Editing

Research has displayed that exhausted CD8+ T cells from cancer patients express high levels of three NR4A transcription factors, which are initiated by NFAT [169,170]. CAR-T-cells lacking all three NR4A transcription factors display increased cytokine production, gene expression associated with effector CD8+ cells, and increased survival and tumor regression in tumor-bearing mice, with chromatin motifs exhibiting enhanced binding at nuclear factor kappa-light-chain-enhancer of activated B cells (NF-kB) and AP-1 sites [169]. The AP-1 transcription factor has proven to be an additional target exhibiting therapeutic potential, as engineered overexpression of c-Jun, a subunit of AP-1, leads to increased CAR-T-cell expansion and antitumoral effects, and decreases in terminal differentiation and exhaustion [170]. Analyses of AP-1, NF-kB, and NFAT show that AP-1 and NF-KB activation prevent exhaustion and aid in T-cell persistence, yet increases in NFAT levels indicate T cell dysfunction [171,172,173]. Recent research has implemented a gene fusion between caspase recruitment domain-containing protein 11 (CARD11) and phosphoinositide-3-kinase regulatory subunit 3 (PIK3R3) to achieve elevated levels of NF-kB and AP-1 without elevation in NFAT expression [173]. These transcriptional changes resulted in increased CAR-T-cell secretion of IL-2 and decreased levels of PD-1, indicative of the expansion and persistence without exhaustion [173]. As discussed above, other approaches have conferred steroid resistance in CAR-T-cells by zinc finger nuclease-mediated disruption of the glucocorticoid receptor, further highlighting the utility of gene editing to improve the efficacy and persistence of CAR-T-cells [17].

The combination of CRISPR-Cas9-based gene therapy and synthetic T cell biology have been used to develop “universal” off-the-shelf clinical products that have improved persistence, effector function, and patient accessibility [174]. CRISPR-Cas9-targeting of checkpoint inhibitors (PD-1, CTLA-4, LAG-3) [105,175,176], activation-induced cell death ligands (FasR/FasL) [177], and immunosuppressive mediators (DGK) [178] may result in improved CAR-T-cell persistence and effector function in vivo. Further, the use of CRISPR-Cas9 for CAR-T generation and manufacturing may drastically improve availability and accessibility for patients. There are many limitations when deriving CAR-T-cells from autologous (patient’s own) T cells, including a low yield and health of peripheral T cells from patients previously treated with chemotherapy and radiation. The use of allogeneic T cells (donor) presents a robust and healthy source of T cells but often results in graft-versus-host disease (GvHD) [179]. To address this, groups have placed the CAR construct expression under the control of the T-cell receptor alpha constant (TRAC) locus, which knocks out the endogenous TCR, addressing the issue of GvHD [180,181]. These approaches, among others, may lead to new generations of CAR-T products that have both improved function and safety.

## 6. Antigen Heterogeneity and Off-Target Toxicity

A significant contributor to the aggressive nature of GBM lies in its inherent intra-tumor heterogeneity [182]. This heterogeneity is typified by clonal and subclonal differentiated tumor cell populations, glioma stem cells, and various constituents within the TME [183] **(**Figure 1E).

Due to this heterogeneity, CAR-T-cell therapy, which only targets one or a few antigens, is inefficient for treating the whole tumor population [184]. Antigen escape is one major reason why clinical trials are not showing substantial efficacy for CAR-T-cell therapy in GBM [185]. Focusing on multiple targets and targets with more consistent expression patterns may enhance the efficacy of tumor eradication. Yet, it concurrently elevates the risk of on-target off-tumor toxicity to a considerable degree. In the subsequent section, we will briefly discuss prospective treatment modalities to mitigate this challenge, which are presently being evaluated in preclinical trials.

### 6.1. Targeting Multiple Antigens

Combination therapy of CAR-T cells with T cell-engaging antibody molecules (TEAMs) presents a promising method to combat GBM antigen heterogeneity and antigen loss in growing tumor cells, allowing for the recognition of these cells by CARs employing single or tandem antigen recognition strategies. TEAMs are antibodies that link two single-chain variable fragments, with one fragment targeting a specific antigen, while the other binds the T cell CD3 receptor [186,187]. Two preclinical studies laid the foundation for the INCIPIENT trial discussed previously [24]. In this trial on the efficacy of CAR-TEAM cells against GBM, EGFRvIII-targeting CAR-T cells were engineered to secrete TEAMs targeting the wild-type EGFR receptor, as antigen loss in tumors treated with EGFRvIII CAR alone is correlated with tumor persistence and amplification of wild-type EGFR surface expression [188,189]. Employment of the CAR-TEAM combination resulted in increased recruitment and directed killing of bystander effector T cells against GBM tumor cells, promotion of the effector memory T cell phenotype, decreased expression of surface exhaustion markers, and effective killing of multiple EGFRvIII-EGFR+ and EGFvIII+ glioma models and patient-derived xenografts [188,189]. Preliminary results from the first patients indicate that the tested CARv3-TEAMs have manageable side-effects. Interestingly CAR-T-cells found in the blood showed less than 2% of surface-bound TEAM, while in the CSF samples surface-bound TEAM varied between 17.6% and 56.2%, indicating that TEAM-E may allow safe and local targeting of wildtype EGFR in the CNS.

Furthermore, the previously mentioned SynNotch CAR-T-cells may allow targeting more universally expressed tumor antigens while preventing off-target toxicities. A SynNotch receptor that detects a specific priming antigen, such as the heterogeneous but tumor-specific EGFRvIII or the CNS tissue-specific antigen myelin oligodendrocyte glycoprotein (MOG), can be used to locally induce expression of a CAR as demonstrated by Choe et al. This enables controlled tumor cell killing by targeting antigens that are homogeneous but not absolutely tumor-specific [165].

### 6.2. NKG2D Ligands

Incorporation of the NKG2D (natural killer group 2, member D) receptor into CAR-T and CAR-NK cell therapies presents a novel, MHC-independent mechanism to achieve effective GBM killing. The NKG2D-CARs possess an NKG2D extracellular domain and a CD3 intracellular domain, with various designs incorporating CD28 or 4-1BB co-stimulatory domains [190]. The NKG2D receptor is expressed on the surface on NK cells, NKT cells, γδ T cells, and CD8+ cells and directly interacts with the NKG2DL ligand, whose expression is elevated on the glioma cell surface [190]. Notably, recent preclinical work displays that CD8+ T cells achieve specific killing of glioma cells completely lacking in MHC-I expression through the NKG2D–NKG2DL interaction; however, these T cells require activation through recognition of their cognate antigen, which can be presented via myeloid cells in the surrounding TME or by neighboring MHC I+ glioma cells [191]. There are currently three clinical trials (NCT04717999, NCT04270461, NCT04550663) incorporating NKG2D into CAR-T-cells for killing NKG2DL+ glioma [192]. NKG2D-targeted therapy is currently hindered by the fact that tumor cells will express a high level of soluble NKG2DL in place of surface NKG2DL, which results in the desensitization in NKG2D effector cells and impaired antitumoral activity [192,193,194].

### 6.3. Ligand-Induced Degradation/Degrons

Various strategies have emerged to develop “suicide switches,” such as inducible caspase-9 [141,195]. Though useful, this strategy results in the irreversible inhibition of CAR-T activity. Newer approaches, such as ligand-induced degradation (LID) allow for reversible on/off modulation of CAR activity in response to the clinically approved drug lenalidomide [196,197,198]. Recently, Kim et al. reported the use of degron-based bioPROTACs for CAR signaling modulation [199]. Similar to SynNotch and SNIPR, bioPROTACs are genetic circuits that are coupled to protein degradation [200]. bioPROTACs are comprised of multiple protein domains: one that binds to a protein of interest and another that induces ubiquitination via E3 ligases or short degron sequences. Kim et al. show the use of a bioPROTAC to degrade ZAP70, a necessary component of CAR-T signaling. Such studies highlight the importance of designing sensitive and modular safety switches for future generations of CAR-T-cell therapy which may reduce both toxicities and T cell exhaustion.

## 7. Conclusions

GBM constitutes an aggressive tumor associated with a dismal prognosis. Consequently, a compelling imperative exists for exploring innovative therapeutic interventions. CAR-T-cell therapy has garnered substantial attention within the realm of immunotherapy, showcasing promise across various tumor types, including GBM. Over time, refinements in the structures and functionalities of CAR-T cells have been pursued to enhance their efficacy in targeting tumor antigens. Nevertheless, the formidable challenges posed by the immunosuppressive TME, inter- and intra-tumoral heterogeneity, the immune-privileged status of the brain, and the hypoxic conditions prevailing in the tumor environment present formidable hurdles in the development of efficacious CAR-T-cell therapies for GBM. Nonetheless, novel innovative therapeutic strategies have emerged, such as blood–brain barrier opening with low-intensity pulsed focused ultrasound or the advent of SynNotch CAR-T-cell therapy. Although these strategies are yet to undergo validation in clinical trials, they hold the promise in enhancing the presence, persistence, and potency of CAR-T-cell therapy, offering hope for future breakthroughs in GBM therapy.
